# A Comprehensive Review on Adaptability of Network Forensics Frameworks for Mobile Cloud Computing

**DOI:** 10.1155/2014/547062

**Published:** 2014-07-06

**Authors:** Suleman Khan, Muhammad Shiraz, Ainuddin Wahid Abdul Wahab, Abdullah Gani, Qi Han, Zulkanain Bin Abdul Rahman

**Affiliations:** ^1^Centre for Mobile Cloud Computing Research (C4MCCR), Faculty of Computer Science and Information Technology, University of Malaya, 50603 Lembah Pantai, Kuala Lumpur, Malaysia; ^2^University of Malaya, 50603 Lembah Pantai, Kuala Lumpur, Malaysia

## Abstract

Network forensics enables investigation and identification of network attacks through the retrieved digital content. The proliferation of smartphones and the cost-effective universal data access through cloud has made Mobile Cloud Computing (MCC) a congenital target for network attacks. However, confines in carrying out forensics in MCC is interrelated with the autonomous cloud hosting companies and their policies for restricted access to the digital content in the back-end cloud platforms. It implies that existing Network Forensic Frameworks (NFFs) have limited impact in the MCC paradigm. To this end, we qualitatively analyze the adaptability of existing NFFs when applied to the MCC. Explicitly, the fundamental mechanisms of NFFs are highlighted and then analyzed using the most relevant parameters. A classification is proposed to help understand the anatomy of existing NFFs. Subsequently, a comparison is given that explores the functional similarities and deviations among NFFs. The paper concludes by discussing research challenges for progressive network forensics in MCC.

## 1. Introduction

The latest development in IT has introduced the concept of mobile cloud computing (MCC), in which data are stored and applications are processed in computational clouds. MCC is employed to mitigate problems related to battery life, computational power, memory capacity, and processing delays in smartphone devices [1]. Specifically, computationally intensive applications are offloaded to the computational cloud, which executes and returns the results back to the smartphone device [2]. These applications are executed on remote resources, such as physical and virtual machines, provided by cloud service providers (CSPs). Users are unaware of the location where the offloaded applications are executed. This condition implies that the execution of applications is transparent owing to the concept of virtualization in MCC [3–5]. However, no process in MCC is possible without network links that connect resources within and outside the cloud. Such network communication links in MCC are called “network positioning,” which is divided into three types, namely, cloud access, data center, and intercloud networks [6]. All network positions in MCC are subject to network attacks that affect various hosts, servers, and data centers. Attackers access network links and perform malicious actions on network packets to propagate adverse effects to cloud resources. Eavesdropping, data modification, IP address spoofing, DoS, DDoS, man-in-the-middle, and packet content modification are examples of such attacks [7]. Researchers have proposed several network forensic frameworks (NFFs) to explore digital evidence and identify the origin of attacks [8, 9], detect malicious code [10], and monitor attackers' activities in traditional networks [11]. However, MCC networks lack NFFs, which are necessary given the number of attacks that occur in MCC networks.

Attackers access cloud resources through cloud access networks and perform malicious actions inside the cloud [12]. A comprehensive approach is required to investigate such malicious behavior by extracting legal evidence from various network devices and network positions in MCC, which is only possible when a network forensics investigation (NFI) has access to the networks of MCC. NFI has access only to the cloud access network and not the data center and intercloud networks in MCC [13]. Such limitation restricts the capability of NFI to investigate attacks and identify evidence found in the networks inside the cloud. To address this issue, CSPs must perform their own network forensics and identify legal evidence. Such approach would provide forensics as a service (FaaS) [14] to MCC users [15]. A number of current NFFs can help CSPs adapt to MCC networks to identify vulnerabilities and the origin of the attack [16–18]. However, comprehensive studies on the adaptability of current NFFs to MCC networks are rare. To our knowledge, no study has focused on the implementation and adaptability of current NFFs to MCC network infrastructures.

This study is motivated by the difficulty of addressing numerous attacks [15] and the lack of NFFs for MCC networks. It focuses on three different aspects of forensics for MCCs, namely, (a) adaptability of current NFFs to MCC networks, (b) provision of FaaS to MCC users, and (c) use of current NFFs to convey information on malicious attacks in MCC networks with only a few false-negative results. Two reasons explain this selection. First, this selection saves time and cost that CSPs might spend on reinventing the wheel regarding NFFs. Second, this assortment narrows down the scope of network forensics in MCC for a comprehensive study of the subject. The objective of this comprehensive review is to provide researchers with insights into the latest ideas in current literature, adaptability of current NFFs to MCC networks, and unresolved issues and challenges faced by CSPs. Such review is critical given that calibration and depiction of legal influences have yet to be considered by CSPs and legislators.

The following are the contributions of this study.

- Classification of current NFFs based on their implementation.
- Identification of evaluation parameters for current NFFs based on the MCC context.
- Analysis of current NFFs based on evaluation parameters that highlight similarities and differences among NFFs.
- Identification of issues and challenges in deploying NFFs to investigate cybercrimes in MCC.

The paper is organized as follows.  Section 2 presents the theoretical framework by explaining MCC, digital forensics, and the significance of network forensics and positions in MCC.  Section 3 presents the overview and classification of current NFFs based on their implementation and explains the anatomy of each current NFF.  Section 4 presents the qualitative analysis of current NFFs based on selected evaluation parameters in the context of adaptability to MCC. Existing NFFs are also compared to highlight the similarities and differences of their adaptability to MCC networks.  Section 5 discusses related issues and challenges.  Section 6 provides the conclusion and future research directions.

## 2. Background

This section provides background knowledge for a comprehensive understanding of the rest of the paper. Digital forensics is introduced, and its process models and role in MCC are examined. MCC is also discussed in terms of its three entity models, namely, smartphone devices, networking, and cloud infrastructure, to provide readers with knowledge on each part of the MCC infrastructure. The importance of network forensics in the MCC paradigm is also discussed by identifying the significance of NFFs for MCC networks in detecting various vulnerabilities in such networks.

### 2.1. Mobile Cloud Computing

**Figure 1:**
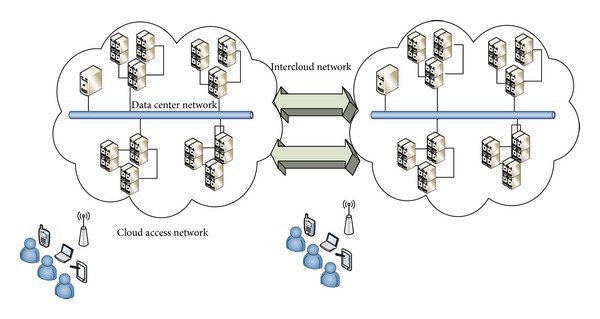
Mobile cloud computing basic network architecture.

MCC is a revolutionary model that allows mobile users to connect to computational clouds through their smart mobile devices from anywhere at any time [6]. MCC is a combination of smartphone devices, wireless channels and network links, and clouds, as shown in Figure 1. Smartphones that connect to MCC suffer from constraints, such as limited battery life, memory, and processing unit as well as delays in executing an application. Such constraints prevent users from executing computation-intensive applications on a lightweight smartphone device [1]. To overcome these problems, different offloading mechanisms [19–22] have been proposed to partially or entirely outsource the computational load of smart mobile devices.

Cloud computing also involves the combination of various resources to form data centers; users can then utilize data center resources to compute and store applications [23]. These resources are integrated by the CSP in data centers that merge to form a cloud [24, 25]. Clouds help smartphone users connect to the Internet from any location. Users can connect to cloud resources at any time; CSPs integrate the resources of different organizations and provide a virtual environment to facilitate smartphone use in MCC [6]. Users benefit from the virtual resources assigned by CSP as they experience reduced time delays, increased availability and reliability, and proper load balancing in computation-intensive applications. In addition, users are only charged by CSP for what they have used in the cloud [26]. A cheaper means to utilize the powerful resources of MCC without paying for their infrastructure is thus provided. Cloud resources have high storage capacities, which are utilized by smartphone users to transfer large amounts of important data to MCC. Storing data in the cloud prevents data loss, virus attacks, leakage, and data alteration when an attacker gains access to a smartphone device [6]. Many service providers, such as Amazon S3 [27], Drop Box [28], Google Drive [29], Google Docs [30], and http://www.SalesForce.com/ [31], allow users to store their data in a cloud. These cloud services can be accessed by connecting to the Internet through different networks.

Smartphones connect to 3G/4G or wireless networks, which serve as a gateway to cloud services. These networks are accessible and allow smartphone users to utilize network services that connect them to MCC from any location. Smartphones also have integrated Wi-Fi chipsets that connect such devices to wireless networks, which are also called 802.11 networks [1]. A smartphone can join a wireless network when it is within range of an access point to detect the signal. A wireless network is the simplest and most inexpensive means to connect smartphones to MCC via the Internet. However, using wireless networks to connect to the Internet and MCC presents certain constraints related to power assignment [32], load balancing [33], channel assignment [34], and secure communication [35]. Moreover, wireless communication involves weak encryption techniques [35] and thus allows an attacker to exploit networks mostly through DoS and DDoS attacks. Many smartphone users are unaware of such attacks and the vulnerability of wireless networks and are thus likely to be victimized by various attackers.

The MCC model is composed of different types of networks, including cloud access, data center, and intercloud networks. However, data center and intercloud networks are inaccessible to NFI in its investigation of various cybercrimes that occur in an MCC network. Security breaches in MCC networks require comprehensive NFFs to overcome various network susceptibilities. The probability of an attack increases as networks continually evolve, and the network forensics process must be mandatory to identify and prevent cyberattacks. The following section explains the fundamentals of digital forensic mechanisms.

### 2.2. Digital Forensics

Digital devices are vulnerable to security breaches [36]. Hackers employ malware and spyware to exploit the security flaws of smartphone devices [37]. Malware and spyware are malicious codes that allow an attacker to spy on user activities through smartphone data, such as email, calls, Internet browsing, SMS, and GPS location. Attackers eavesdrop on users' conversation through malware and spyware software [37]. Digital forensics is employed to investigate cyberattacks and malicious codes that affect digital devices, particularly smartphones devices in the MCC paradigm. Evidence from digital artifacts is identified to investigate the malicious behaviors of attackers [38], including monitoring, altering, deleting, inserting, and copying user data on digital devices. The malicious behaviors of attackers compromise the confidential credentials of users by damaging their privacy [39]. Several models of the digital forensics process have been proposed to conduct digital investigation in different research aspects, such as law enforcement, military operations, and business and industry [38, 40–45].

**Figure 2:**
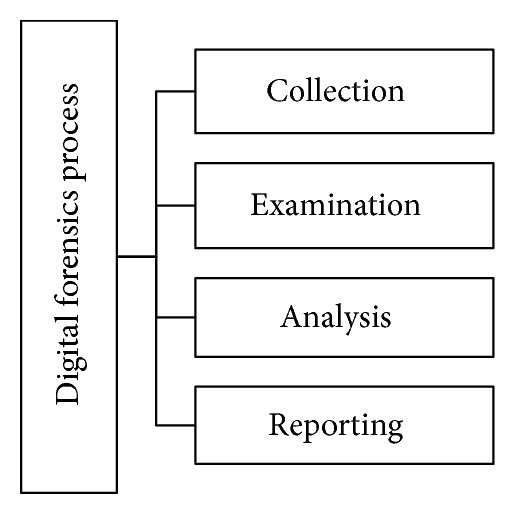
Digital forensics process model.

A digital forensics process model requires four steps to conduct the investigation; these steps are acquisition, identification, evaluation, and admission. These steps are integrated to obtain digital evidence from digital artifacts, which are then presented in court [46]. Alternatively, these steps were modified into five steps, namely, preservation, collection, examination, analysis, and presentation, in [40]. The first harmonized digital forensic investigation model was developed by integrating existing iterative and multitier models [47] to perform efficient digital investigation under legal terms and conditions. The National Institute of Standard and Technology explains the digital forensic process in their report by proposing four steps, namely, collection, examination, analysis, and reporting, as shown in Figure 2 [48].

The collection phase is the initial stage, wherein digital evidence is collected from digital artifacts. This stage is crucial because collecting incorrect data in this step results in errors in the subsequent stages. The examination phase focuses on the significance of the data to the digital investigation process. The data collected are stored in a memory storage device or a portable drive that allows data to be transferred without altering the format and losing the integrity of the data [49]. The next phase analyzes the data to identify various vulnerabilities and the malicious behavior of the attacker as well as determine the origin of the attack. The analysis is performed with several digital forensic tools, such as EnCase [50], FTK [51], Sleuth Kit [52], and Helix [53]. These tools identify digital evidence extracted from temporary, deleted, and register files as well as from cache, cookies, email, and metadata present in digital devices [54, 55]. Such digital evidence acts as proof against attackers in court by presenting a complete and accurate investigation report. The investigation report is the final phase of digital forensics. All the activities performed by forensic investigators in the preceding stages present a complete picture of the investigation process in terms of a legal document.

Digital forensics is fast becoming a hot topic in MCC. The demand for digital forensics in MCC continues to increase because of the increasing number of attacks, such as DoS, DDoS, and botnets [5, 15, 56, 57]. The origin of attackers must be investigated to stop these attackers from performing further malicious acts. Such investigation in MCC requires a comprehensive digital forensics strategy to perform data collection and examination through virtual situations, live forensics, evidence segregation, and proactive preparation mechanism [58]. The rapid progression and increasing attractiveness of MCC necessitate the establishment of digital forensics in the cloud environment. Novel investigation techniques are required to prevent cybercrimes in MCC. However, several challenges in MCC, such as access to the entire cloud, jurisdictional issues, and technological advancement, create problems in forensics investigation [13]. Nevertheless, such situation also creates opportunities to establish standards, policies, and procedures in cloud-based forensics.

### 2.3. Network Forensics in Mobile Cloud Computing

Cloud services in MCC are acquired by smartphone users by connecting through Wi-Fi, WLAN, 3G/4G, and long-term evolution networks [1]. These networks have to be fast and secure enough to send user requests to computational clouds and send the results back to smartphone users. However, these networks are targeted by attackers to gain access to the network in the form of a network breach [59]. The current security solution involves the use of intrusion detection systems (IDS) and firewalls to detect and identify attack patterns [12]. However, intelligent attacks circumvent such security solutions to propagate malicious activities in the network [60]. Thus, security solutions should be sufficiently intelligent to detect intelligent attacks that compromise the system [61].

The network forensics process identifies attacks while monitoring and analyzing network traffic. Network forensics delivers two types of services in the investigation process. First, it detects intrusion and malicious traffic [61]. Second, it collects and analyzes network traffic by performing traceback [16], using attack graphs, and parsing voice over IP (VoIP) [62] in a converged network. Collecting network traffic from high-bandwidth network channels restricts network forensics from capturing large amounts of network traffic, particularly in MCC. High-bandwidth networks pass millions of network packets per second and require a large storage capacity to store network packets for further analysis. One option is to use the storage services of cloud data centers to store and access large amounts of data [63]. Large amounts of data are mostly generated in smartphones through the use of multimedia data applications, such as video chat (Skype), video uploading or downloading (YouTube), audio traffic (iPlayer), and online games. Most of the multimedia traffic are based on real time and must flow quickly and securely to perform accurate communication. To capture multimedia traffic, live forensics technique [64] is required to identify vulnerabilities in the network flow on a real-time basis. Live forensics technique is utilized to capture volatile network traffic that can be eliminated with the power off or on arrival of new network traffic. Volatile data must be captured in network communication for two main reasons. First, network packets pass through different ports and reach their destination without being stored at the destination. Second, attackers reach the compromised system, collect confidential data, and delete trace outs. Network forensics must be conducted at live data communication to address these problems [65]. However, this approach incorporates the overheads of highly computational processors, I/O devices, and large storage devices for collecting, analyzing, and storing real-time network traffic. Such overheads are minimized by collecting data only at peak hours when capturing attacks is also likely to be achieved [66]. The processing time and storage load can then be reduced, and precise results can be obtained in the forensics investigation. The rest of the network links that are inaccessible for real-time network investigation has to be searched to identify attack patterns that would assist in tracing the origin of the attack.

The significance of network forensics is in each part of the MCC's network communication channels. Malicious behavior in network packets needs to be traced through NFF whether a smartphone user is connected to mobile clouds or data centers are interconnected or linked to other cloud data centers. NFIs have limited access to investigate different network susceptibilities [13]; thus, network forensics should become a permanent service to MCC users through cloud network channels and resources.

### 2.4. Network Positions in Mobile Cloud Computing

Network position shows the location of the network that connects two entities in MCC. Three types of networks generally exist in MCC: cloud access, data center, and intercloud networks [6], as shown in Table 1. Cloud access network is the network that connects smartphone devices and the cloud through the Internet [67]. This network is utilized when a smartphone user connects to the cloud to offload and download an application to the cloud [76]. However, connection difficulties related to security, compliance, privacy, and high availability exist. The cloud itself is composed of data centers, and a data center is a combination of resources. The network that connects all resources within a single data center or the network that combines one or more data centers is called “data center network,” which in turn involves intra- and interdata center networks [69]. Data center networks are employed in the cloud when data are transferred from one resource to another or to other data center resources within the cloud for execution, such as for cluster computing. Scalability and cost effectiveness are the two issues related to this network [69]. Scalability depends on the architectural design, and cost effectiveness depends on the power consumption of a data center network [77].

The connecting network between two or more clouds is called intercloud network [70]. Such network deploys a fiber optics network that has high bandwidth and high-speed line rate. Intercloud network is used when one cloud migrates or sends an application to another cloud for execution or storage. Intercloud network provides a dedicated network and increases transfer speed through protocol optimization. A brief description of each network position in MCC is presented in Table 2. All network positions in MCC are considered vulnerable to attacks in NFFs. No network is safe from an attack because of vulnerabilities, which require further investigation to determine the origin of the attack [8].

Network forensics has a vital function in investigating networks to identify legal evidence on cyberattacks [16]. Network attacks performed on traditional networks can be easily investigated by obtaining legal evidence through current NFFs [71]. Such networks are accessible and allow for immediate investigation. However, data center and intercloud networks in MCC are inaccessible to third-party NFIs because these networks are the sole property of CSPs. The lack of third-party access limits the investigation of NFIs on cloud networks [13]. The best option is for CSPs to conduct their own network investigation and provide FaaS to users, which also generates additional revenue. Almost all networks within the cloud territory are accessible to CSPs; network attacks can be easily investigated, and legal evidence can be obtained. Moreover, MCC users would find it easy to trust the data and believe that such data are safe from third-party NFIs [72].

## 3. Network Forensic Frameworks

The classification of NFFs based on an exhaustive literature review is presented in the first part of this section. Such classification is derived from the implementation of the architectural frameworks of network forensics, which narrows down the scope and allows for a comprehensive study of the area. NFFs are classified into five categories, namely, traceback NFFs, converged networks, intrusion detection systems, attack graphs, and distributive frameworks.

In the second part of this section, the structural aspect of current NFFs is discussed in detail by reviewing its frameworks, approach, methods, evaluation, limitations, and output performance. A complete operational overview of each NFF and its implementation objectives is presented.

### 3.1. Overview and Classification of Network Forensics Frameworks

Network forensics aims to identify legal evidence from network traffic to investigate the origin of the attack and attacker behavior [8]. NFFs capture and analyze network traffic in the network to investigate attacks performed by different attackers [16]. NFFs extract information from network traffic to rebuild emails, messages, FTP traffic, and various other communications. The process helps NFIs reconstruct the attack path and determine the attack's origin [62]. Traditional networks can readily access breached network devices and acquire data for investigation. However, accessing and acquiring legal evidence from breached devices in MCC are difficult because of the CSPs' resource property and virtual resource infrastructure [3]. NFIs are unable to initiate the investigation process without access to network resources in the cloud. Network data integrity in MCC is another concern [49]. Anyone can access cloud resources and alter user data, resulting in the loss of data integrity. Frequent data migration within various clouds can also affect data integrity because of the storage of data in different formats and in different databases. Privacy is also an issue in the investigation of MCC networks because user information could be disclosed when a malicious activity is investigated [72]. User data traveling in the network at the same time further complicates efforts to capture malicious user data among various users, especially in high-speed data rate networks in MCC. Each network link might contain millions of user data simultaneously. Thus, identifying a specific user's data without disturbing that of the others is difficult. Real-time analysis is also a challenging task in MCC because the NFI cannot access cloud networks [74]. Such analysis is important because network data are volatile. Network data might lose their identity after being overwritten by other data, and closing the session could allow attackers to alter the data and delete attack traces from the network. In business, health, and other industries, real-time analysis is necessary to handle malicious situations occurring in the environment. Current network forensic tools lack the capability to capture, record, and analyze high-speed line rate data at various channels of MCC networks [58, 75]. An intelligent network forensics tool that is compatible with the virtual and disseminated environment of MCC networks is thus essential. Moreover, high bandwidth is necessary to acquire legal evidence from different locations in MCC [73]. Acquiring data from a remote cloud data center network requires a dedicated bandwidth to execute the process quickly and respond to user queries in real time. Traditional networks are simpler than MCC networks because of the former's access to resources; such access allows for the use of less bandwidth with quick incident responses. A chain of custody for network evidence is also important for the MCC environment. Each event in the MCC network has to be validated, particularly as to how and where the data are stored on cloud resources while maintaining their integrity [75, 78]. Chain of custody has to be defended in court to present evidence against the attacker. Tracing each event while preserving its integrity and reliability is a challenge because of virtualized and distributed environments in MCC and the incorporation of data migration. A comparison of the status of issues regarding current network forensics and MCC network forensics is presented in Table 3. A comprehensive review is required to explain the aforementioned problems in current NFFs and illustrate deliberate compatibility with MCC networks.

NFFs can be classified into five categories, namely, traceback NFFs, converged networks, intrusion detection systems, attack graphs, and distributive frameworks. This classification is based on an exhaustive literature review. Traceback NFFs [8, 9, 16, 71, 85] identify the origin of the malicious packet in the network generated during a cyberattack. Traceback NFFs are utilized to determine the source of the packet generation [86] and eventually lead to the identification of attackers based on their source IP address. Moreover, NFFs in converged networks distinguish evidence from audio, video, and multimedia data [62, 87, 88]. Converged networks are prone to attacks because of their flaws in communication [89, 90]. In this study, NFFs for converged networks are only demonstrated for VoIP traffic. NFFs identify evidence from voice packets altered by attackers during their attack on the network. Intrusion detection systems (IDSs) are also employed to detect and log malicious packets generated by attackers [91–94]. IDSs are utilized to check packets based on provided rules and knowledge obtained by performing pattern matching at the time of capture. Packets are logged through hash functions and sent to the forensics server for further analysis. Furthermore, NFFs employ attack graphs to identify attack paths in the network. Attack graphs help reconstruct attacks by determining the activities performed by an attacker during the attack [17, 95–99]. Such a graph refines the attack scenario by studying its sequential steps from the origin of the packet to the victim node in the network. Distributive NFFs are utilized to identify malicious packets in disseminated networks by capturing them from different locations [18, 100–102]. Distributive NFFs eliminate the bottleneck problem, which often results in high bandwidth consumption and long delays.  Table 4 shows the classification of NFFs. The function of each NFF is illustrated.

### 3.2. Structure of Network Forensics Frameworks

This section discusses the structure of NFFs based on their approach, method, evaluation, limitations, and performance. The approach attribute presents the techniques employed by NFFs to conduct the investigation. Such techniques include logging [8], packet marking [9], spread spectrum [85], probabilistic model [17], dynamic forensics intrusion tolerance [92], forensics examination [97], distributive network forensics [100], and visualization and interaction [99] for various NFFs.

Logging is utilized to record network traffic and its patterns in the form of log files to obtain evidence on various attacks [8, 16, 18, 62, 71, 87, 88, 93, 94, 101, 102]. This technique captures network traffic at different routers and performs a hashing function on it. Log files are usually applied by a hash function that validates the files' integrity; such files can be accessed later for the investigation of several attacks. Packet marking is an approach utilized to mark packets at the router to trace packet movement in the network, which is vital for tracing back the origin of an attack [9, 71, 101]. Packet marking is commonly adopted by traceback frameworks [9, 71] to identify the attacker's source address, which they often spoof. Spread spectrum techniques are employed to traceback the origin of the attack, detect attacks, and identify receiver status by spreading signals with a frequency in a domain of a bandwidth [85]. A probabilistic model measures the uncertainty present in the attacked networks to obtain digital evidence [17, 91]. Dynamic forensic intrusion tolerance performs in a situation where a forensics server is affected by an attacker [92]. It provides real-time tolerance to the server and analyzes network traffic. Forensics examination is implemented to examine log files altered by attackers during an attack to guard against investigation [97]. Distributive network forensics collects network logs from distributed agents in disseminated networks and analyzes the logs locally or centrally through forensics servers [100]. Visualization and interaction approach makes the entire attack graph easy to analyze and allows network investigators to examine various attack paths through human-computer interaction interfaces [99].

The method attribute demonstrates how different approaches are employed to identify evidence in the network. Similar to the approach attribute, each NFF uses certain methods as shown in Table 5. Authenticated evidence marking scheme reduces the overhead in the entire network performance by parsing data at edge routers; the authenticity and integrity of network data are thus improved [71]. Lightweight IP traceback method utilizes the time-to-live (TTL) packet field of an IP header to trace packets for investigation [9]. Scalable network forensics traces back the origin of the attack based on attack traffic separated from normal traffic in traces provided over a long period of time [16]. Hopping-based spread spectrum traces back cybercrimes in anonymous network communication by providing security and accuracy to such communication [85]. The IP traceback method employs a sinkhole router, a compressed hash table, and data mining approaches for network forensics analysis to determine the origin of the attack [8].

VoIP forensics network patterns are utilized in VoIP network traffic to collect and analyze voice packets systematically. The technique helps identify, detect, and trace attacks by generating patterns for such attacks [87]. VoIP network forensics also identifies digital evidence on various attacks by comparing normal and abnormal packets of the network. Such method reduces human intervention by collecting voice packets through sensors installed at various parts of the network [88]. A VoIP evidence model reconstructs attack events by applying secure temporal logic of action (S-TLA+), which works when it lacks sufficient information to investigate an attack [62]. This model also provides reliability and integrity for the collected information, validates the authenticity of the provided evidence, and allows NFIs to capture unknown attacks undetected by other network forensics methods.

Probabilistic discovery and inference method is utilized by IDS to reveal unknown information related to the analysis of network traffic [91]. The method is only applicable to network-based IDS and not to application-based IDS. Such probabilistic method aids in forensics explanation, which is based on unreported signature rules and observed network-based IDS alerts. Moreover, formal methods are employed to show that a forensics server is sufficiently tolerant and thus works even if it is subjected by several attacks [92]. The availability of the forensics server is enhanced by collecting significant evidence. Steganography is also utilized to conceal original log files from an attacker. Logs are converted into images and are hidden from the attacker. The conversion is performed by changing the least significant bit for each pixel without being visible to the naked eye [93]. The original log files are kept in the custody of NFIs, which then trace attacks by monitoring copied log files for alteration. Network forensics architecture is applied to perform multidimensional forensic analysis based on log messages and network data [94]. Network traffic is monitored at the time of network entry, and log data are recorded and analyzed as data move out from various security devices. This twofold monitoring and analysis technique ensures the reliability and credibility of evidence extraction through network traffic.

Scalable analysis [95] is performed to measure the effect of current and future attacks in large-scale networks. Large amounts of network traffic are analyzed in real time by measuring the effect of a single attack on the enterprise and its correlation to other attacks [95]. Moreover, a multilevel and multilayer attack tree identifies the system level risk by analyzing various security threads resulting from various network attacks [96]. It helps NFIs become familiar with future network threats. Antiforensics method can be incorporated with attack graphs to trace attackers based on their activities. Antiforensics method urges attackers to perform malicious actions while being monitored [97]. In addition, a fuzzy cognitive map can be incorporated with a genetic algorithm to identify the worst attack path among a large number of attacks present in the network [98] and help NFIs track and reveal attacks that have the worst effect on the network. Raven architecture is utilized to visualize and interact with attack paths in the attack graph [99]. The situation of attack graphs is simplified; thus, NFIs can easily understand several attack paths in the network.

Likewise, distributive architecture is utilized to investigate network packets according to the IP connections, port records, and various session creations between hosts; bloom filter tracking is adopted in this architecture to collect evidence against attackers [100]. This investigation procedure provides comprehensive information against attackers by extracting evidence from various events in the network. Other distributive network forensic architectures are utilized to produce rapid responses in the generation of the attacks while investigating network traffic and logs dynamically [101]. The problem of static analysis is addressed, and time delays in incident response are reduced. Client server architecture is utilized to identify potential risks, misbehavior of packets, and origin of the attack [102]. It captures data from distributed locations, converts traffic into a database, analyzes network attacks, and produces visual reports by performing a statistical analysis of the entire process. The dynamic network forensic model simplifies the situation by providing authentic, integral, and valid digital evidence collected from distributed locations in the network [18]. Agents installed at distributed locations capture raw network traffic and apply antigenic presentation coding on it. This condition helps NFIs collect real-time evidence on network attacks.

The evaluation attribute in Table 5 represents the techniques employed by NFFs to analyze frameworks. Different NFFs employ various methods to evaluate the framework. These methods include test bed [71, 88, 91, 97], simulation [8, 71, 85, 98], tree analysis algorithm [9], S-TLC+ [62], finite state machine [92], prototyped [93], synthetic & real attack graph [95], case studies [96], and many other scenarios [17]. The limitation attribute reveals the shortcomings of NFFs that affect the entire network forensics process as the investigation is performed. These shortcomings include computational overhead [71, 94, 95], storage overhead [71, 92, 96, 101], router overhead [8, 9], capturing real time network traffic [16], scalability [85, 87, 93, 96, 97], time consuming [88], bandwidth utilization [88], forensics server bottle neck [18, 87, 102], observation depended [98], lack of awareness [98], and specific attack investigation [100]. Table 5 also presents the output derived from evaluating NFFs based on their evaluation values in the performance attribute.

## 4. Review of Network Forensics Frameworks in the Context of Adaptability to MCC

This section highlights the parameters used for evaluating adaptability of existing NFFs in MCC networks. The parameters include scalability, overhead, accuracy, complexity, and privacy. Most of the NFFs discussed in the review deal with capturing the network packets from networks, followed by the analysis phase on forensics servers. In case of large distributed networks, forensic servers should scale to analyze huge amount of network traffic. In particular, existing NFFs must support network forensics by collecting network evidences from various distributed networks connecting millions of MCC resources. Therefore, NFFs must scale in order to collect, preserve, analyze, and report network evidence in MCC in a real-time. The overhead of existing NFFs is required to be minimal in terms of (a) computation and (b) storage. Computational resources are required to analyze huge amount of networked data to extract sources of evidence. On the other hand, this data is also stored in a system that has to be analyzed later by various forensics mechanisms. The accuracy attribute for NFFs is vital to measure filtration of irrelevant network traffic for analysis of evidence. Extraction of significant data from the network is important in MCC due to restricted access to the cloud data. High accuracy results in less time for analysis of data and producing quick incident response. The complexity of existing NFFs can be analyzed for MCC networks in terms of its implementation, collection, investigation, and analysis. It implies that it is difficult to apply existing NFFs in MCC networks due to inaccessibility of cloud network, virtualization, and distributed networks. The privacy is considered as one of the most important issues for MCC nowadays. User migration towards the cloud must ensure the data integrity and safety from unauthorized access. Privacy in NFF is significantly different as compared to the MCC. Therefore, applicability of NFFs in MCC must be evaluated in terms of privacy.

### 4.1. Evaluation Parameters for the Analysis of NFFs

*Scalability* is an important MCC parameter [103]. Scalability is enabled by the concept of virtualization in different entities, such as servers, data centers, resources, operating systems, and networks. Scalability in terms of networks maximizes throughput, improves performance, and ensures availability and reliability [104]. Increased network scalability enhances data mobility, which ultimately demands more monitoring and analysis features in the context of network forensics. Therefore, NFFs are a vital part of MCC to investigate network links and devices that scale according to the demands. Moreover, current NFFs [16–18, 99] can integrate features with attack patterns and security devices to investigate network traffic for MCC. Scalability can be regarded from two perspectives: horizontal and vertical scalability for MCC [105].

*Horizontal scalability* is also called “scale out”; it deals with system throughput by enhancing its complexity. Horizontal scalability increases the number of counts in terms of hardware resources and performs tasks more quickly [106]. The scope of horizontal scalability for MCC is mainly a result of disseminated networks that combine different data centers within and out of the cloud. The capacity of network forensic servers has to be sufficiently large to capture, record, and analyze network traffic from several network links and devices in the cloud environment. For instance, one forensic server may be used for one trillion packets instead of using two or more forensic servers for the same number of packets for investigation [92]. Sending network packets through the shortest path to their destination reduces the number of hop counts and thus helps NFIs easily capture and analyze network packets with less time delay and increased system throughput.

*Vertical scalability* is known as “scale up.” It handles the improvement of the existing functions and features of a system by adding more hardware [105]. For instance, adding extra processors or memory in a network forensic server executes and stores more network packets with less time delay. Similarly, network speed and bandwidth can be enhanced to provide an efficient response to MCC user queries. NFFs attain efficiency by investigating more network packets within the same time frame. However, upgrading network resources does not always produce sound results because of diminishing returns that increase with a decrease in the performance rate. For this reason, one has to identify other factors to incorporate with scalability to produce effective network forensic outputs. Nevertheless, the vertical scalability of NFFs in MCC is important because of its rapid incident response to identifying the criminal behavior of attackers at the time of the attack. Most current NFFs lack such scalability, which needs to be adopted to meet the objectives in MCC [8, 71, 87, 88, 93, 94, 96, 97].

*Overhead* is related to the sophistication of NFFs and reduces the performance of the system. Network forensic overhead includes computational and storage overheads. These two overheads are calculated for MCC based on existing NFFs. The computational overhead of NFFs includes network processing, bandwidth delays, packet marking, preserving, analyzing, collecting data, investigating, logging, and integrating overheads [8, 9, 71, 97]. These factors are incorporated into the computational overhead attribute that degrades the computation of the system. The storage overhead of NFFs deals with network traffic storage at different locations and devices in the disseminated networks of MCC [8, 71].

*Computational overhead* (Co) is high for NFFs in MCC when a framework utilizes many resources for its computation, employs a less reliable investigation mechanism, and addresses irrelevant data, resulting in a time-consuming analysis [8, 9, 71, 87, 92, 93, 97]. The value for Co is low for NFFs in MCC when a system utilizes minimal resources to analyze network traffic packets [62, 85, 88, 91, 95, 96, 98, 100–102]. The value is moderated by utilizing resources that both increase nor decrease system performance and throughput in the computation [16–18, 94, 99].

*Storage overhead* (So) is ranked high when it does not have a proper mechanism to store large dispersed network packets at different locations and devices for several networks in MCC [8, 71]. Network traffic is mostly monitored and captured at different network security devices, which suffer from small buffer space to store large and high-speed data network packets. However, the value for So is low and decreases drastically when a dedicated physical resource is assigned near the cloud to store entire logs of the network traffic [16–18, 87, 92–96]. Such store logs can be further sent to forensic servers placed in the cloud for investigation depending on the architectural framework of NFFs to manage high network traffic in the disseminated networks of MCC.

*Accuracy* of NFFs in MCC is calculated by separating irrelevant data from large amounts of network traffic. The accuracy of NFFs is high when network traffic is filtered to investigate the rest of the traffic; otherwise, the accuracy is low. Network traffic that enters the network incorporates two types of data traffic, namely, normal and abnormal or infected data traffic [16]. NFIs are highly concerned about abnormal data traffic because it contains evidence on attacks and the behavior of attackers. Reducing or separating normal data traffic from abnormal data traffic increases accuracy and helps investigators perform their investigation on a specific network traffic with minimal time delay [16].

However, many NFFs [18, 71, 87, 94, 97, 102] capture the entire network data traffic and thus result in delays and decline of system performance. To overcome these problems, an investigation must be performed on relevant data depending on the situational requirements. Extracting relevant data is also a challenging task for NFIs, particularly in high-speed data rate networks of MCC.

Consequently, the accuracy attribute value will be high for NFFs if the maximum level of irrelevant data is separated, such as separating normal data traffic from abnormal data traffic, without utilizing additional resources and reducing system performance [16, 98]. The easiest means is to use cloud computational resources because these are computation-intensive and have more processors to execute the process rapidly. However, the value of NFFs is marked low when NFI is performed on the entire network traffic found on various networks links; this condition ultimately results in time delays and reduced system performance [18, 71, 87, 94, 97, 102]. The value is marked moderate when network traffic is reduced, allowing for the analysis of an attacker's behavior within a reasonable range of time delay and system performance [8, 9, 17].

*Complexity* attribute of NFFs illustrates the problem faced by NFIs in conducting network forensic investigations in the MCC environment. The network forensic process consists of sequential steps performed in the current network infrastructure. Such steps include collection, examination, analysis, and reporting [48]. Current NFFs also incorporate architectural implementation complexity in MCC because of its proposed frameworks. These frameworks have to be adaptive for several networks in MCC to reduce the complexity faced by NFIs.

However, adapting current NFFs in MCC is a challenging task. The process must be flexible in terms of virtualization and distributed characteristics of MCC. In addition, the mobility characteristics of data in clouds have increased the complexity of tracking, collecting, and analyzing such data. Several types of network positioning, such as cloud access, data center, and intercloud networks, also affect the complexity of current NFFs. Thus, the complexity of current NFFs is high in collecting [18, 88, 96, 100], analyzing [9, 17, 18, 62, 87, 88, 91–102], and investigating network data [16] as well as in the implementation of NFFs in MCC [8, 18, 62, 71, 85, 87, 88, 91, 92, 94, 96, 97].

*Privacy* is one of the important factors that divert users toward MCC. Smartphone users execute and store data in clouds to protect their data from various attacks. User data reach cloud resources by passing through cloud access, data center, and intercloud networks. Network positioning in MCC becomes a battlefield for NFIs in extracting evidence against various attackers [56]. However, confidential user information could become compromised while accessing several networks of MCC. The possibility of user privacy being compromised makes users hesitant to use MCC.

A win-win solution must be established for current NFFs so as not to compromise user privacy while performing investigation on several network positions in MCC. The value of privacy is high for NFFs when user data are not accessed during an investigation; however, this condition remains a challenge, particularly in MCC [99]. The value of the privacy attribute is moderate when some user data are accessed during the investigation [8, 9, 17, 96–98]; the value is low when user data are accessed during the investigation in MCC [16, 18, 62, 71, 87, 88, 93–95, 100–102].

*Adaptability* parameter can show whether current NFFs are applicable for network positions in MCC or not because of certain constraints. MCC networking has diversified into current networks as a result of its connectivity to millions of servers, capability to transfer trillions of packets per second, stateless computing, dynamic application provisioning and positioning, and virtualized features [107]. MCC networks have to redefine their features to improve their management, scalability, and administrative aspects compared with current NFFs.

However, the adaptability of current NFFs must be measured from the perspective of MCC given its architectural framework, scalability, privacy, accuracy, complexity, overheads, and implementation aspects. These requirements can be met by identifying the differences between current and MCC networks to improve current NFFs with value-added features and make them adaptive for MCC networks. None of the current NFFs is completely adaptable to the MCC network because of the different operational requirements and on-demand services of MCC [8, 88, 94, 98, 102]. The value of the adaptability attribute is defined as “difficult,” “low,” and “moderate” in Table 6. Compatibility between NFFs and MCC networks is nearly impossible to achieve (difficult), but with a few changes in the frameworks and added features, the possibility could improve (moderate) [16]. Nevertheless, adaptability values are marked low when compatibility is between difficult and moderate; this condition reveals the need for major changes to adapt to MCC networks. In most cases, NFFs lack scalability [8, 71, 87, 88, 93, 94, 96, 97], accuracy [62, 85, 88, 91–93, 95, 96, 99–101], and privacy to enhance their adaptability to MCC networks [85, 91, 92].

### 4.2. Analysis of Existing Network Forensics Frameworks to MCC

This section presents a comprehensive analysis of existing NFFs in terms of their adaptability to MCC networks. Current NFFs are investigated based on the selected evaluation parameters discussed in Section 4.1.

#### 4.2.1. Traceback Based Forensics Frameworks to MCC

The network forensic evidence acquisition (NFEA) framework [71] lacks scalability in terms of MCC because of packet capturing at edge routers in current networks. Identifying edge routers is difficult in MCC because of the seamless connectivity provided by CSPs to cloud users [108]. Determining and accessing the appropriate edge router are challenging tasks for network forensics in MCC. Moreover, computational overhead is high because a three-phase encoding scheme that collects, encrypts, and marks each packet at the edge router is utilized. This scheme requires computational resources to perform encoding for a large number of network packets, especially in MCC. Computational resources are managed in the cloud and accessed through the pay-as-you-go service model. Storage overhead is also high because no technique is incorporated to manage high network traffic. Storage resources in the cloud are utilized to store large numbers of marked network packets. However, payment is required to access the services and resources of computational clouds; thus, utilizing cloud resources is expensive. Accuracy is also low in terms of MCC because NFEA employs a flow-based selection marking scheme to categorize network traffic based on IP attributes [109]. However, network traffic is reduced, resulting in efficient management of large data in MCC. NFEA frameworks are highly complex in terms of implementation. Applying a three-step encoding scheme for each network packet at various network locations in the cloud is difficult. The privacy attribute is low because each packet is captured and marked at the router, which discloses important information on users instead of the attacker. Consequently, reliability decreases, and the number of users is reduced. In conclusion, adapting the NFEA framework for MCC networks is difficult given the abovementioned problems.

Lightweight IP traceback scheme (LWIP) [9] incorporates horizontal scalability with an increasing number of hop counts. Each router inserts a TTL value in the packet field to simplify the investigation process. Large networks have numerous routers that receive packets by setting the TTL value. This feature of LWIP is scalable enough for MCC networks but results in a high computational overhead as a result of analyzing the packets with the tree analysis algorithm utilized by LWIP. The analysis requires time frames with computational resources and thus increases the computational overhead, particularly with trillions of packets flowing through MCC networks. Storage overhead is at a moderate level because LWIP only stores packet information in terms of a packet package, which is utilized to tally with IP addresses. A router only marks the TTL field of the packet and does not store it. LWIP is also moderately accurate because it involves a filtration step to remove irrelevant packets and only mark packets that are a threat. LWIP complexity is high in the analysis phase of MCC because of the tree analysis algorithm. The algorithm requires sufficient packets to reconstruct the path with more time frames, which is difficult because of the seamless connectivity and real-time incidence response requirement in MCC [110]. LWIP reaches a moderate level of privacy because of the marking of packets at the TTL field rather than at the payload field. All these constraints make the LWIP framework difficult to be adapted for the MCC network.

Scalable network forensics strategy (scalable-NF) [16] adopts vertical scalability resulting from the improved computational power of the forensics server because only infected traffic is investigated. MCC network data can be investigated by analyzing only infected network packets rather than the traffic flows of the entire network. However, the computational overhead is low because of the training phase that generates a normal behavior profile from network traffic through probabilistic inference. Such generation requires computational resources that can be extracted with MCC resources in the cloud. A normal behavior profile contains a set of features with their values that can be stored in different storage resources provided by MCC. Thus, the storage overhead for scalable-NF is low in MCC. The accuracy of scalable-NF is high because infected packets are separated from normal packets, which is favorable for MCC networks. However, the complexity level is high because random moonwalk algorithm is employed in forensics investigation. This algorithm regards the directed host graph as an input and then investigates attack edges. Generating attack graphs and identifying attack edges are difficult in MCC given its virtualized and distributed environment with frequent service and data migration [2]. The privacy parameter is low in MCC because network traffic is divided into normal and attack traffic (infected), both of which require a thorough analysis of the packets. Therefore, scalable-NF can adapt to MCC networks by improving the analysis phase and increasing the privacy of user data.

Hopping-based spread spectrum technique (HB-SST) [85] provides horizontal scalability in spreading a spectrum over a number of nodes. Large networks can utilize this technique through secure dispersion of spread codes in both time and frequency domains. The computational overhead is moderate owing to the sending of pseudonoise codes with a large number of packets in large networks, particularly in MCC. Computational overhead can be reduced by utilizing MCC resources according to the demand. However, implementing such a framework in MCC is complex because of the use of pseudocodes in high-speed data rate networks that connect data centers, servers, and network devices in different clouds. In MCC, data migrate within numerous devices that require a quick response when an attack occurs in real time. However, storage, accuracy, and privacy parameters are inapplicable to MCC networks because there are no concerns over the storage and reduction of data in HB-SST. Therefore, adapting this technique to MCC network positions is extremely difficult.

IP traceback protocol (ITP) [8] lacks scalability because of its architectural implementation. ITP has a high computational overhead owing to the large message passing among system, router, data base, and attack analysis managers. Such message passing among various managers increases network latency utilization, which further increases the overall investigation time. The router manager detects an attack packet sent to the sinkhole router that stores and sends it to the system manager. This process creates high storage overhead at the sinkhole router and causes a bottleneck vulnerable to attacks. Each packet is treated at more than one place, resulting in high complexity, particularly for a large network infrastructure such as MCC. Complexity increases when trillions of packets enter the network. Each router then performs static functions, such as hashing, compressing, storing, and diverting packets toward the sinkhole router. ITP is moderately accurate because data are stored in a compressed hash table format by applying filtering to remove irrelevant data. ITP privacy is at a moderate level because only packet headers are targeted for investigation. An attacker can use packet headers to attack networks by altering different packet fields inside the packet. Therefore, adapting ITP for MCC networks with the current proposed framework is difficult.

#### 4.2.2. Adaptability of Converge Network Based Forensics Frameworks to MCC

Pattern-based network forensics (PBNF) [87] incorporates limited scalability because of its framework implementation. The computational overhead is high because evidence is collected from different sensors installed before VoIP components and network forensic analysis tools are utilized to collect raw network traffic data. Collecting such data and sending them to a centralized forensics server consume network bandwidth and creates traffic load at the server. Analysis is performed in real time, and high bandwidth is required to generate an incident response at runtime. Collecting data also requires high storage capacity. PBNF stores voice data at numerous locations but still requires sufficient storage space to address large amounts of data in MCC. Cloud storage resources are suitable for storing voice data that can be further investigated by a forensics server. Thus, the storage overhead generated by capturing voice data in MCC is low for PBNF. Voice data are captured and analyzed for different attack patterns; this procedure decreases user communication privacy. The privacy factor is low for PBNF because the voice packets of a user are monitored and investigated at different locations in a converged network. The complexity level is high in terms of implementation and analysis because data are captured from multiple locations, the monitoring scheme is inefficient, and large volumes of data are analyzed. Thus, the adaptability of PBNF is ranked as difficult for networks in MCC given the aforementioned limitations.

VoIP network forensic analysis with digital evidence procedure (VoIP-NFDE) [88] lacks scalability because of the deployed analysis method. However, the computational overhead is moderate in terms of differentiating normal from abnormal packets in VoIP network traffic. Such differentiation of packets requires computational resources that can be obtained from MCC on a pay-per-demand basis. The computational overhead in traditional networks is high because of the lack of computational resources; however, the computational overhead in the MCC network infrastructure can be reduced by utilizing cloud resources. Comparing trillions of packets per second at multiple locations in MCC requires a large storage capacity for the storage of captured network data without losing their integrity. VoIP-NFDE has a moderate level of storage overhead because MCC storage resources are utilized at a financial cost. Furthermore, network traffic is not reduced by filtering; thus, the VoIP-NFDE framework in MCC lacks accuracy. The complexity of this method's implementation, collection, and analysis in MCC is high because of the static nature of the framework. The privacy level is low because differentiating the packets to expose malicious ones increases the risk of leaking out user information. Thus, VoIP-NFDE is difficult to adapt to large and fast disseminated networks of MCC.

The VoIP evidence model (VoIPEM) [62] contains horizontal scalability in terms of collecting information from different VoIP components in a converged network. The model can be utilized for large network infrastructures by providing information from various VoIP components at the cost of high complexity in implementation and analysis as well as low privacy of user data. Implementation complexity results from the synchronization of VoIPEM modules that collect and send information to generate an evidence module. Analysis complexity is high because of the different scenarios inferred from forward and backward chaining performed by S-TLC modeling. The computational overhead is moderate because evidence from attack scenarios is generated through S-TLC. However, storage space is required to store infected packets, from which a hypothesis is formulated to identify unknown attacks. VoIPEM lacks a proper storage mechanism for traditional networks; however, the model can use the storage resources of the MCC infrastructure. Moreover, VoIPEM does not focus on reducing any type of network data. Hence, its accuracy is inapplicable to both traditional and MCC networks. VoIPEM should thus improve its strategies on computational overhead, accuracy, and privacy to become adaptable for MCC networks.

#### 4.2.3. Adaptability of Intrusion Detection Systems Based Forensics Frameworks to MCC

Analytical intrusion detection framework (AIDF) for distributive IDS [91] includes horizontal scalability in terms of distributed IDS to detect intrusions by generating attack alert messages. AIDF performs by inhibiting negative behavior in which an attack is detected as distributive but fails to report a message. AIDF scalability can be heightened in MCC by installing IDS at various locations in the network to detect malicious behavior in network packets. Enhanced scalability increases complexity in MCC because of the installation of distributive IDS sensors, collection of attack patterns, and real-time performance analysis. However, AIDF has a moderate computational overhead because of its probabilistic inference that identifies hidden undetected attack patterns for better forensics explanation. Probabilistic inference increases delays when an ignorant sensor identifies matching signature rules from its neighboring sensors. The method incorporates storage overhead because of its storage requirement at the distributed IDS, large network traffic at specific sensor nodes, and storing of network traffic of all sensor nodes at the central location. The AIDF storage overhead with regard to all sensor nodes is at a medium level and high when a single sensor handles a large amount of network traffic. However, the storage overhead can be minimized by using MCC storage resources. Accuracy and privacy parameters are inapplicable to AIDF because it does not reduce network traffic for investigation and employs probabilistic inference for analysis. Therefore, adapting to MCC networks is difficult for AIDF given its existing infrastructure.

An intrusion tolerance system for modeling and analyzing dynamic forensics system (DFITM) was proposed in [92]. The system achieves horizontal scalability by employing two servers to receive packets. Scalability is increased by installing more forensic servers to analyze infected packets for legal evidence in MCC. However, DFITM has a high computational overhead because it employs a formalized method to investigate network packets. DFITM results in more time delays and requires computational resources, especially in a network that contains thousands of nodes, such as MCC. The storage overhead is low as a result of storing network traffic in a normal server and the use of infected network packets in a shadow server to store malicious packets that were investigated to identify the origin of the attack. Storage overhead can be minimized by using MCC storage resources to store all infected packets without overwriting them. However, DFITM has high complexity in terms of its implementation and analysis in the MCC context. The system employs multiple steps to perform analysis. Such steps include generating a security report through the shadow server; the report is sent to the evidence collector and further partitioned, encrypted, and replicated in various evidence databases. Accuracy and privacy are inapplicable to MCC because network traffic is not reduced and the analysis of network packets results in the lack of user privacy. Assessing thousands of nodes, large network channels, and trillions of packets per second as well as the virtualization environment of MCC limit the adaptability of DFITM to such an environment.

Intrusion investigation framework with data hiding schemes (IIFDH) [93] lacks scalability because of its architectural framework. A monitoring module is utilized to monitor trace log files altered by attackers. In MCC, the framework requires numerous monitoring modules because of the millions of trace log files present at disperse locations. IIFDH also lacks accuracy because it does not filter network traffic and consequently increases delays. The framework has a low computational overhead for traditional networks and a high computational overhead for MCC networks. In MCC, additional working processes are required to embed log sources, trace log files, perform stenography, monitor alteration in the traces, and create backup files in log backups for numerous locations. The storage overhead is high for traditional networks and low for MCC networks. Storing original trace log files in log backups can be easily performed through cloud storage resources. IIFDH has high complexity because of the stenography performed on trace log files by changing the least significant bit. This task can be more challenging in MCC, which can have millions of log files at numerous locations in the cloud. Privacy is low for user data in MCC because monitoring log files can leak out confidential user data. Therefore, IIFDH is difficult to implement for MCC networks because of the nonscalable framework, lack of accuracy, minimal privacy, and high computational overhead of the employed framework.

Network forensics based on intrusion detection analysis (NFIDA) [94] lacks scalability because of its architecture, such as the use of network evidence-capturing engine and network forensics analysis engine. These engines work in sequence to produce digital evidence based on log messages and network data. The engines can be disadvantageous to large networks that require real-time investigation of high-speed data rate network traffic at various locations in the cloud. NFIDA has a high computational overhead in traditional networks as a result of its encryption method during both network data and log message capturing. In MCC, the computational overhead can be reduced by using computation-intensive resources for encryption. The storage overhead is high in a traditional network owing to the network traffic burden on the centralized network forensics analysis engine. The network forensics analysis engine receives log messages and network traffic. Nevertheless, the storage overhead in MCC can be minimized by using storage resources to store log messages and network data. NFIDA has low accuracy because it omits irrelevant data through the network forensics analysis engine. Moreover, NFIDA is complex due to its implementation and analysis phase in MCC. User data privacy is low in NFIDA because packets have to be investigated using the network forensic analysis engine. Making NFIDA adaptable to the MCC network infrastructure is difficult. NFIDA has to be modified in terms of its scalability, accuracy, and privacy before it can be applied to MCC networks.

#### 4.2.4. Adaptability of Attack Graphs Based Forensics Frameworks to MCC

Scalable analysis approach (SA) [95] achieves high horizontal scalability in terms of addressing large amounts of raw security traffic. SA can address millions of nodes present in a network by determining their timespan distribution and dependency on one another. However, in a traditional framework, SA has a high computational overhead, such as timespan distribution and probabilistic temporal attack graph that requires computational resources to generate computation results for attack modeling. However, the computational overhead can be reduced by assigning cloud resources. SA has a medium-level storage overhead in traditional networks when the attack and dependency graphs increase; a portion of the graphs is stored on the disk rather than in the main memory. The storage overhead can be also minimized by storing a portion of the graphs on cloud storage resources. The complexity of SA in MCC is high when millions of nodes are addressed because the virtualized and distributed setup creates difficulties in analyzing various attack paths in the attack graphs. SA compatibility is low in terms of its adaptability to MCC, and the accuracy factor is inapplicable because the entire network is focused on creating the attack graph. The framework of SA requires modification with regard to accuracy and complexity prior to its application to MCC networks.

Network attack modeling based on multilevel and layer attack tree (MLL-AT) [96] lacks scalability and has a medium level of computational overhead in terms of constructing attack sequences composed of various single attacks. MLL-AT requires computational resources to determine attack sequences and can use cloud resources in the case of MCC. The model has low storage overhead because it only stores attack sequences; storage overhead can be easily minimized by using the storage resources of MCC. Data are not reduced for evaluation and modeling; thus, accuracy is inapplicable to both traditional and MCC networks. MLL-AT has high complexity in terms of implementation, collection, and analysis to address attack sequences in attack trees. MLL-AT is more complex in MCC because it locates risk values for attack sequences, which can be difficult because millions of nodes are present in the MCC network. The method has a medium level of privacy because it has minimal access to user data given that it only identifies the attack sequence. Such privacy level is acceptable for MCC users who wish to keep their information safe from investigators. MLL-AT has a low level of adaptability to MCC with its current framework setup. However, it can be enhanced by modifying its strategies to increase its accuracy and reduce its complexity so that it can be applicable to MCC.

Attack graph for forensic examination (AGFE) [97] lacks the scalability factor because it inserts anti-forensics nodes in the attack graph; this condition results in sophistication owing to the incorporation of anti-forensics nodes into millions of nodes for large networks, such as MCC. AGFE has a high computational overhead because it inserts antiforensics nodes into the attack graph to trace out unexploited attack behavior. Antiforensics nodes are inserted into the attack graph that incorporates computational overhead as a result of the dependency among various nodes. In MCC, a cloud integrates millions of nodes connected with high data rate network links; millions of packets are transferred per second. To trace such nodes, antiforensics nodes should be sufficiently powerful to trace high-speed data rate traffic within the seamless and virtualized connectivity provided by MCC. An antiforensics database also involves overhead that can be solved by utilizing the storage resources of MCC. In addition, AGFE reduces the number of network nodes that are not utilized in the forensics examination. AGFE results in low accuracy because not enough network traffic is filtered. Complexity is high in terms of implementation and analysis stages because of the insertion of the antiforensics nodes and the determination of interdependency among network nodes in MCC. Privacy in AGFE is at the maximum level because investigators are provided access to data. However, the method lacks scalability; otherwise, it is highly adaptive to MCC networks.

Fuzzy cognitive map (FCM) [98] achieves horizontal scalability by reducing a large number of attack paths to identify the worst one. FCM is useful for MCC, which has a wide range of network links that connect millions of resources. In MCC, the large number of network nodes and paths has to be reduced to identify the worst attack paths in the network and consequently help investigators in their investigation. FCM minimizes the number of attack paths through a genetic algorithm (GA) that identifies the worst attack paths in an attack graph generated for the network. FCM analyzes only relevant attack paths and thus increases accuracy in identifying the worst attack paths in MCC. FCM has a medium-level computational and storage overhead because it employs fuzzy cognitive map processing and stores attack graphs for the network. These attack graphs are further utilized to investigate the worst attack paths in the network. FCM can reduce both computational and storage overhead by using MCC computational and storage resources that are lacking in traditional networks. However, the complexity of FCM is high because of its analysis phase for MCC. The method generates cognitive maps by developing concepts and casual influence for large network nodes; this condition makes the entire process extremely complex. In addition, FCS has a medium level of privacy because user data are not observed and only attack nodes are targeted to develop attack graphs. Such privacy level is suitable for MCC networks to keep user data confidential from third-party investigators. Overall, FCM is adaptive to MCC networks; however, its complexity must be minimized further, particularly when worst attack paths are investigated in real-time scenarios.

Probabilistic approach to identify cost-benefit security hardening (CBSH) [17] incorporates horizontal scalability to perform risk assessment of nodes in large networks. The objective is to determine the cause of an attack in large networks with a large number of nodes. The scalability of CBSH is applicable to the disseminated network of MCC because it assesses risks to identify the cause of an attack. CBSH has low computational and storage overheads because of the application of probabilistic method to perform risk assessment for the cause of the attack without storing an entire network node for investigation. Computation-intensive resources are employed to perform probabilistic inferences, and storage resources are utilized to store targeted attack nodes. However, CBSH accuracy is ranked as medium in the MCC aspect because it fails to reduce all the irrelevant network nodes while performing probabilistic inferences. Privacy is also ranked as medium because it investigates target attack nodes without inferring user data, which increases confidentiality and privacy for users. CBSH employs analysis phase complexity by performing cost-benefit security hardening for large disseminated networks in MCC. Thus, CBSH is applicable to MCC networks; however, reducing unwanted network nodes is necessary for the model to perform real-time probabilistic inferences and produce a quick incident response to various queries.

Visualization and interaction framework for attack graphs (AGVI) [99] contains horizontal scalability in terms of visualizing attack nodes for large networks and helps investigators interact with a visualized interface. Such interface of visualized attack graphs enables investigators to search for a specific attack path in the attack graphs. Visualization and interaction are useful for large networks that contain millions of nodes, especially for MCC. AGVI also provides a facility for visualizing attack paths between two selected vertices on an interface and thus helps investigators during the investigation process. AGVI complexity is high in intercloud networks that should generate an attack path in the network in real-time situations. The model has low complexity in cloud data center networks. The level of privacy is high for users in the MCC infrastructure because AGVI provides visualized attack graphs for infected nodes of the network rather than accessing user data. However, accuracy and storage overhead factors are inapplicable to AGVI in MCC because the network nodes are not reduced and stored. Therefore, AGVI provides more benefits to service providers in their visualization of attack paths in MCC networks.

#### 4.2.5. Adaptability of Distributive Forensics Frameworks to MCC

Distributed framework (ForNet) [100] follows vertical scalability in terms of a highly computational forensics server in MCC. In ForNet, synApps software module is installed at different network devices to collect network traffic and send it to the forensics server. The forensics server should be computation-intensive to investigate network traffic with a quick incident response in real-time situations. This condition can be easily achieved with related MCC resources. ForNet has a medium-level computational overhead because of synApps, which has to be fast enough to cope with fast-moving network traffic. The framework also has medium-level storage overhead because network traffic is diverted toward a centralized forensics server and has to be stored in its database for the investigation of various network vulnerabilities. However, ForNet lacks accuracy because it investigates all network traffic without any reduction through synApps installed at various locations of the network. The complexity of ForNet is viewed in MCC as a result of its collection and analysis phases. Collection is performed with synApps modules that should be capable of capturing entire network traffic while preserving its integrity and reliability for sending to the forensics server. ForNet cannot be applied to collect network traffic from disseminated parts of MCC and cannot be investigated centrally by the forensics server because it causes numerous time delays, low incident responses, and reduction of the entire system performance. Nevertheless, the privacy level is low because all network traffic is captured and investigated centrally by analyzing each of its packets to identify various vulnerabilities. Capturing all network traffic from dispersed network devices and investigating them centrally is a difficult task. The ForNet framework requires modification prior to application in MCC networks. ForNet can be applicable for MCC networks if it contains distribution storage with decentralized analysis of captured network traffic to reduce time delays, provide a quick incidence response, and increase system performance.

Distributed agent-based real-time network intrusion forensic system (DRNIFS) [101] employs both horizontal and vertical scalability. Horizontal scalability is achieved by installing network agents at the sensitive areas of a large network. Vertical scalability is achieved by providing a highly computational forensics server at disseminated locations. DRNIFS helps collect network traffic from dispersed areas in MCC and provides a quick incident response for queries by performing investigation at various forensics servers. However, DRNIFS has medium-level computational and storage overheads in terms of collecting and storing data that are to be further investigated to identify malicious behavior and reconstruct various attacks. DRNIFS includes log and audit data, network traffic, and a historical network of misused patterns. The computational and storage resources of MCC can be utilized to minimize these overheads and make DRNIFS adaptive for MCC networks. Collecting and investigating various types of data result in high complexity because various data in distributed locations, particularly in real-time situations, are analyzed. The process is time consuming and can delay responses, which are significant in real-time investigations. Additionally, complete network traffic is captured and investigated by various forensics servers; thus, user privacy is reduced. DRNIFS also lacks accuracy in MCC networks. DRNIFS is partially applicable to MCC networks and can be improved by enhancing its accuracy and privacy parameters for network traffic that contains multiple users' information.

Distributed cooperative network forensics model (DCNFM) was proposed in [102]. This model implements horizontal scalability because of its forensics servers. These servers are distributed in the network, collect network traffic, and store data from various client agents in a database format. Several forensics servers are added to the network according to its requirement. To investigate the same type of network traffic (e.g., e-mail traffic), only one network forensics server is required. Although DCNFM can utilize computation-intensive cloud servers, it requires numerous servers because of the millions of data resources and high network traffic in MCC. DCNFM has a medium level of computational overhead because the process involves multiple executions, such as building databases, filtering and dumping traffic streams, converting traffic streams to a database format, mining forensics databases, network-surveying, and visualizing network attacks. The model has storage overhead because it captures the entire network traffic to reconstruct the attack behavior. This storage overhead can be easily addressed in MCC by storing the entire network traffic in multiple storage resources to prevent the data from being overwritten. However, capturing the entire network data reduces the accuracy level of DCNFM in MCC. Nonetheless, the level of accuracy is medium when the model focuses on specific types of network traffic, such as email data. This condition can be achieved at the filter and dump stage of the DCNFM process. DCNFM is complex because it collects and analyzes network traffic in MCC. Validation is required to show that complete data are captured at various security devices in MCC networks. Correspondingly, the analysis performed at multiple locations requires synchronization of the investigated data at different forensics servers to provide a quick response to attack queries. The privacy level is low because entire data are captured and analyzed for malicious behavior; thus, confidential user data could leak out. As a result, DCNFM has low adaptability to MCC and has to improve its accuracy and privacy parameters. Network data collection must be performed in less time, and investigated data must be synchronized in the various forensics servers.

Dynamical network forensics framework based on immune agent (DNF-IA) [18] integrates horizontal scalability in terms of detector agents distributed in the network. Detector agents are spread across the network to collect network traffic and forward it to the forensics server. The number of detector agents varies depending on the network's requirement. This feature can be useful to MCC in locating detector agents in the sensitive area where network data must be recorded. The number of detector agents depends on the size of the network. The framework has medium-level storage overhead in traditional networks because network traffic is stored locally at various detector agents. However, DNF-IA can minimize the storage overhead by utilizing the storage resources of MCC. Similarly, computational overhead is also high in traditional networks because a single forensics server has to investigate millions of packets and send a quick incident response. The abundant computational resources of computational clouds can minimize the problem through fast analysis and investigation with quick incident responses. DNF-IA lacks accuracy because the entire network traffic is captured and recorded and data are not filtered; thus, DNF-IA is time consuming and problematic, particularly in MCC. DNF-IA has low privacy because it analyzes network traffic captured from various detector agents installed at disseminated locations of the network. The model investigates each network packet by identifying malicious codes that can violate user privacy through access to the packets. The complexity of DNF-IA in MCC is the result of the implementation of detector agents and collection of network traffic from various MCC networks. When, where, and how detector agents should be placed in MCC to capture complete and accurate network traffic have yet to be determined. Collecting complete network packets while preserving their integrity at disseminated locations in the MCC network is also an important task for CSPs; this task has to be verified and validated. Overall, DNF-IA could be made applicable to MCC network investigation; however, its functionality must be enhanced to increase accuracy and privacy for network traffic with improved real-time incident responses. Table 6 provides a comparison of current NFFs based on their adaptability to MCC.

NFFs must be modified in terms of their architecture by incorporating different modules and embedding different strategies prior to their adoption in MCC networks. NFF frameworks generally have computational and storage overheads because of the high-speed data rate and large network traffic in current networks [8, 9, 71, 87, 92, 93, 97]. Additionally, NFIs require computation-intensive resources to investigate trillions of network packets in MCC because of the high bandwidth, real-time application support, advanced technologies, and various cloud services provided by MCC to their users. Capturing large amounts of network traffic requires a huge storage space to log network packets to be investigated for malicious behavior through different NFFs [16, 63, 98]. Utilizing cloud resources, such as computational and storage resources, can overcome these problems. The accuracy parameter also serves a vital function in the compatibility of current NFFs with MCC networks. Filtering large amounts of network traffic data allows NFIs to readily analyze the specific data and identify digital evidence against an attacker. The accuracy value is low in most NFFs. This condition shows that NFFs lack a filtering mechanism to reduce network traffic during an investigation and make NFFs adaptable for MCC networks [18, 71, 87, 94, 97, 102]. Incorporating a filtering mechanism into the existing architecture of NFFs to handle high-speed data rate and high network traffic in MCC is the most important factor in determining the adaptability of NFFs to MCC. The complexity of NFFs lies in their implementation, collection, and analysis. Implementing current NFFs for MCC networks is subject to constraints in architectural mismatch, scalability, accuracy, and privacy [8, 18, 62, 71, 85, 87, 88, 91, 92, 94, 96, 97]. The entire system is made sophisticated to produce an efficient output for investigating digital evidence in the various networks of MCC. The collection phase of NFFs does not guarantee complete capturing of network traffic and validation and verification of its integrity [18, 88, 96, 100, 102]. Capturing the entire network traffic at various network locations is important in determining the origin of the attack and the attack behavior. The analysis phase for various NFFs involves investigating the entire network traffic captured from the network and results in numerous time delays with less incident responses to investigation queries [9, 17, 18, 62, 87, 88, 91, 95, 99, 101]. This problem can be minimized by assigning distributed computation-intensive servers at various locations in the cloud. Furthermore, many NFFs lack privacy, which is important for MCC to gain users' trust [16, 18, 62, 71, 87, 88, 93–95, 100–102]. Privacy is low when network traffic logs are accessed; access to traffic logs involves access to other user information in the network, which is against the characteristics of MCC. An intelligent framework requires a multitenant environment of MCC to protect user data from being exploited.

In conclusion, NFFs' computational and storage overhead problems can be solved by utilizing the abundant resources of computational clouds. With these resources, NFFs can store network logs in storage resources and utilize computation-intensive resources to investigate network traffic. Similarly, the scalability of NFFS can be increased by capturing, examining, storing, preserving, and analyzing network traffic at disseminated locations in resource-rich computational clouds. Machine learning and data mining techniques should be incorporated to retrieve relevant data to trace the origin of the attacker and his/her malicious activity. Thus, the accuracy of NFFs in MCC can be increased. Complexity would also be reduced if the aforementioned suggestions and solutions are adopted for NFFs in the context of MCC. User privacy can be increased by adopting artificial intelligence techniques to extract specific user data that have to be investigated accordingly. Such techniques help NFFs become adaptive to MCC networks and identify different vulnerabilities in the network. CSPs will also benefit from the use of current NFFs with slight modifications; FaaS can be provided to users, and additional revenue can be generated.

As discussed earlier, the adaption of existing NFF in MCC is challenging primarily due to the restricted access to the data. It implies that NFF adaption in MCC must depend upon predictive analysis and techniques of artificial intelligence. Artificial intelligence can help in the identification of features that reveal information worthy for analysis. It has been shown that artificial intelligence can be effectively used for information gathering from the distributed networks of the MCC [111]. Explicitly, Artificial Neutral Networks (ANN) can be used to analyze network evidence from various databases and online resources connected through high bandwidth networks. Beside ANN, Support Vector Machine (SVM) can be used to find out the patterns in the MCC networks based upon their classification [112]. The SVM trains the data through various intelligent algorithms to identify patterns in the network traffic to help classify network traffic into categories. Furthermore, forensic in MCC can benefit from swarm intelligence as well [113]. Swarm intelligence can predict network attacks by modeling bioinspired algorithms which are designed to solve complex problems. Moreover, fuzzy logic can also be an option to identify network evidence in huge distributed networks of MCC [114]. The networked data in MCC has significantly increased due to steep rise in mobile users. To tackle the networked data, real-time network evidence mechanisms demand accurate approximations of vulnerabilities through fuzzy logic systems. Thus, it has been observed that existing NFF lacks artificial intelligence mechanisms, which can assist network forensics in MCC networks.

## 5. Issues and Challenges in Network Forensics for MCC

**Figure 3:**
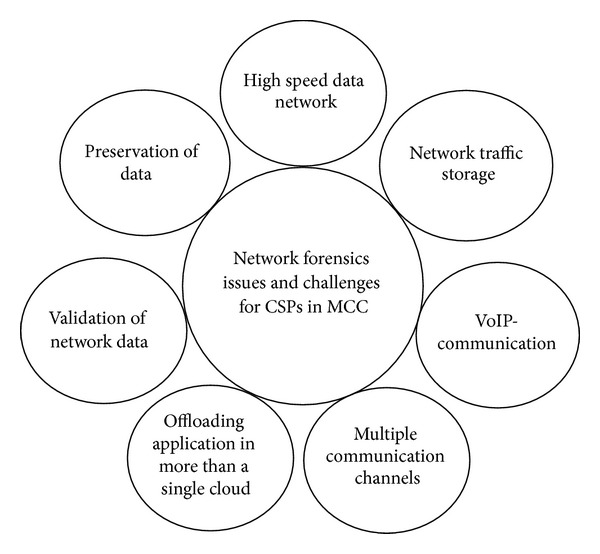
Network forensics: issues and challenges for CSPs in MCC.

This section describes unresolved issues and research challenges in forensics investigation faced by CSPs in MCC. These issues must be addressed to employ current NFFs in MCC networks. Current NFFs would remain unsuitable for MCC infrastructures until robust methods and approaches are engineered, designed, and incorporated into them.  Figure 3 presents the unresolved issues and challenges faced by CSPs in the MCC environment.

### 5.1. High Speed Data Network

Effective network forensics requires capturing, preserving, examining, and analyzing each event on a single device placed in the entire network [115]. Network forensics helps reconstruct, analyze, and track each incident of exploitation in the network. However, the aforementioned stages are restricted because of the network bandwidth and high-speed data rate in large network channels of MCC. In MCC, various data centers are linked with high-speed fiber optics network channels that send millions of packets per second [69]. Various data centers link together to form a cloud, which provides services to users in the form of computation, storage, and others. The challenge for CSPs is to capture millions of packets per second from disseminated locations in the cloud in real time while preserving data integrity and reliability [116]. CSPs have to investigate network packets captured at various locations in the cloud to identify different vulnerabilities and trace the origin of an attack. CSPs experience difficulty in capturing, indexing, storing, and analyzing a large number of packets and producing an incident response to forensic queries in a time span of less than a second. In several cases, data have to be retrieved from other clouds; this procedure further delays the investigation process and reduces the quality of service.

Current network forensic analysis tools are unsuitable for high-speed data rate network traffic and cannot produce efficient results in large cloud networks, such as MCC [50–53]. The challenge is to develop an intelligent network forensic analysis tool that can help CSPs investigate network packets and ensure that FaaS outputs are provided in real-time situations for user queries.

### 5.2. Network Traffic Storage

Large network volumes with high-bandwidth network channels have made network forensics investigation complex and challenging, particularly for MCC networks. MCC is utilized by smartphone users because of data-related constraints [108], such as, integrity, security, preservation, consistency, and storage. Smartphone users send their data to the cloud for storage; they can easily access and retrieve these data any time [1]. Capturing and storing digital evidence with the large amount of network traffic and high-speed data rate in MCC are challenging for CSPs because all these activities are performed in less than a second [117]. Storing network traffic requires a proper storage mechanism that does not affect data integrity and reliability. Moreover, storage resources in MCC should be selected in such a manner that they are readily accessible for retrieving network logs whenever necessary.

Cloud storage resources minimize the storage overhead for current NFFs in MCC; however, they increase the complexity of retrieving particular network records from numerous distributed storage databases [118]. Millions of network packets are considered and stored in the disseminated storage resources of a cloud; large storage capacity and an optimized searching algorithm are thus required [119]. Data mining techniques can be utilized to solve this problem by retrieving relevant network records from large storage databases that can be easily investigated for various vulnerabilities [120]. Therefore, cloud storage resources increase the adaptability of current NFFs to MCC and minimize their storage overhead. However, much effort is required to formalize the network traffic storage process in the cloud to produce standardized and real-time FaaS to MCC users.

### 5.3. Voice over Internet Protocol-Communication

Smartphone users utilize Skype, Viper, and other voice chat applications to communicate with other users. Such applications generate VoIP packets, with high priority on quality of services [121]. VoIP communication must be captured in a real-time manner during a forensic investigation. Forensics investigation that checks for latency, jitter, and packet loss has to be performed dynamically for VoIP traffic to identify the malicious behavior of voice packets [62].

However, CSPs face difficulties in collecting voice packets stored in different places and moved from one server to another in a cloud (e.g., registrar, redirect, location, and proxy servers) as well as in signaling gateways and billing systems. Many problems related to the privacy of users arise when accessing such servers to extract voice packets. These servers can be in other cloud territories, which restricts CSPs from investigating voice packets without prior approval because important user information could leak out and related privacy related are noted down in SLA. The entire process is subsequently delayed, and the probability that voice packets would lose their integrity or would be altered by an attacker increases [122]. Such constraints create problems for CSPs to provide full-fledged FaaS in real time to remote users in the MCC paradigm.

### 5.4. Multiple Communication Channels

Multiple communication channels are the paths available to send data from a mobile device to another destination, such as MCC. These channels increase the throughput of the network by sending and receiving data from and to the user at the same time [123]. CSPs face problems during data investigation when the data are sent from smartphone devices to the cloud using a wireless network with multiple communication channels [124]. CSPs do not have complete access to the network between a smartphone device and the cloud because smartphone users are generally mobile [125]. To provide FaaS to MCC users, CSPs must investigate various vulnerabilities present in cloud access networks, such as the network between a smartphone user and the cloud. However, CSPs depend on ISPs and other network service providers to investigate the cloud access network with multiple communication channels open to various network susceptibilities.

Moreover, data pass through multiple communication channels via more than one network type because of the mobility of smartphone users. Such an issue produces additional complexity for investigators to trace smartphone user connectivity within various networks and perform live forensics for multiple communication channels. In multiple communication channels, a network packet is affected by interference with other wireless networks and results in incomplete capturing of network packets [126]. Incomplete capturing of network packets does not provide a complete picture of the evidence to identify attackers and their attack behaviors. Consequently, trustworthiness and dependability are required by CSPs and network service providers to investigate the situation in real time through the provision of transparent FaaS to smartphone users in MCC.

### 5.5. Validation of Network Data

The combination of data centers forms clouds, which are then assigned by CSPs to MCC users for the storage and execution of various applications. Each data center connects with other data centers through high-speed data rate network channels [69]. Each channel has to transfer millions of packets in a second and has to store them in a reliable storage medium without affecting their integrity. However, network forensics encounters difficulties in developing integrity and escalation among distributed network forensic storages in MCC [127]. For instance, user data are stored on two different data centers at two different clouds via connection through high-speed network links; verifying validity and integrity while accessing data scattered between the two different cloud data centers is challenging.

Data validation is important because forensics investigation is performed based on data stored in the data center to identify the origin of the attack and attacker [128]. Each CSP has to ask permission from other CSPs to investigate network resources for possible network susceptibilities. This condition creates problems for CSPs in freely capturing network evidence from intercloud and other networks in the cloud. Accessing evidence from an intercloud network is restricted by cross-border rules or might be delayed by CSPs by not responding in real time. Sometimes, real-time data are required for an investigation; however, such data could also be unavailable in MCC for network forensics [74]. This condition is important for sectors, such as business and health, where real-time situations with quick and accurate responses are necessary. Hence, the validation of network data in cloud computing is a challenge for CSPs in MCC.

### 5.6. Offloading Application to Multiple Clouds

Highly intensive computational applications in MCC are offloaded for execution to data centers in the cloud that are geographically dispersed through high-speed network links [2]. However, data centers sometimes encounter scarcity of a resource to execute an application; thus, a portion or an entire application is migrated to the nearest available cloud for execution [1]. Increased application load on a data center causes the migration of applications to different data centers. This condition has to be observed in the investigation to identify various attacks. Hence, CSPs face the tough task of capturing legal evidence of an offloaded application from each network link that might not be accessible because of another cloud's boundaries. The only means to gain access to the intercloud network is to obtain legal permission from the CSP, with the risk of being denied because of user data privacy and confidentiality issues.

Real-time investigation technique is required to capture offloaded network packets in other clouds because of the volatile nature of network traffic. When the link between clouds is disconnected, network data are lost and tracing back the origin of the attack becomes difficult without enough evidence in the network.

### 5.7. Preservation of Data

Preservation of network data is as important as collecting, examining, and analyzing network traffic to obtain legal evidence against an attacker. Preserving data in the cloud is significant because it provides long-term continuity and usability of digital records for stored network data [129]. Preservation helps in future investigations and can function as a pattern match rule for security devices to manage various attacks in the future [57]. The challenges that CSPs face in data preservation in the cloud include data increase, tendency of data to be lost, changing rules and regulation, data migration, interoperability in the clouds, and lack of authenticity verification. CSPs must employ a formal method to preserve data in the cloud to provide FaaS to the user on demand [130].

A preservation process is required to ensure the extensive availability of network traffic and assists in digital auditing, accessing logs, confidentiality, indexing, data center footprints, security, and high availability. Therefore, advancements in technology guided by legal requirements are required to preserve network data for network forensics investigation.

## 6. Conclusion and Future Directions

This paper discussed the functions, approaches, and structures of current NFFs. We qualitatively analyzed current NFFs based on selected evaluation parameters in the context of their adaptability to MCC. The findings will benefit CSPs by allowing them to save time and money that might be spent on reinventing the use of novel NFFs for various MCC networks. This study provides motivation to users through newly added services, such as FaaS. Current NFFs involve computational and storage overhead problems because of the limited computing potentials of intermediate network devices. However, MCC utilizes resource-rich computational clouds that can solve these problems and help existing NFFs adapt to MCC networks. Similarly, the scalability of NFFs can be increased by capturing, examining, storing, preserving, and analyzing network traffic at disseminated locations in resource-rich computational clouds. Machine learning and data mining techniques can be incorporated to retrieve relevant data to be investigated and trace the origin and malicious activity of the attacker. Doing so will increase the accuracy of NFFs for MCC. In addition, complexity will be reduced if the suggestions and solutions presented in this paper are adapted for NFFs in the context of MCC. User privacy can also be improved with the use of artificial intelligence techniques to extract specific user data that have to be investigated. Such methods help NFFs become adaptive for MCC networks and identify different vulnerabilities in the network. CSPs will also benefit from using current NFFs with slight modifications; FaaS would be provided to users, and additional revenue would be generated.

We conclude that new research roadmaps and programs are required to overcome the issues and challenges faced by CSPs. Standardized rules, secure reference models, protocols, trust architectures, legal contemplation, technological development, and a global regularity body should be established. These requirements can be achieved by harmonizing the efforts of industrial experts, academic researchers, and investigators under legal entity bodies. Thus, the significance of network forensics will increase gradually and will provide economical and viable solutions for investigators in identifying digital evidence against attackers in MCC networks.

As a future research direction, network forensics as an open research area (particularly for MCC networks) has to be further explored with newly adaptive frameworks that consider user privacy, data integrity, data confidentiality, data segregation, and many other factors. However, these frameworks should be incorporated with newly developed dynamic cloud-based network forensic tools to cope with the MCC infrastructure. Similarly, various security threats and attacks must be identified and studied to achieve optimum results in MCC network forensics.

## Figures and Tables

**Table 1:** Generalized network positions in MCC.

Network positioning	Entities link	Example	Objective	Accessibility
Cloud access network [67, 68]	User ↔ cloud services	Internet, NGN, 4G	Dynamic routing, accessibility to cloud	Possible
Data center network [69]	Data center ↔ data center	Cluster computing	Load balancing, virtualization, intensive computing	CSP
Inter cloud network [70]	Cloud system ↔ cloud system	Cloud resource migration	Cloud collaboration	CSP

**Table 2:** Description of network positioning in MCC.

Cloud access network	Data center network (DCN)	Intercloud network
Connects smartphone user to cloud system through wireless, ratio access network (RAN), 3G/4G and LTE networks [4].	DCN connects application software & cluster of computers within data center [70].	Connects two or more cloud systems for cloud collaboration [68]

Public cloud networking makes availability of network applications to users via internet [71].	Its network expands towards connecting two or more data centers with a single cloud system [70]	It not only connects two cloud systems but provides additional functionality such as data format conversions, network virtualization, service availability, address management, intelligent routing, and efficient security [72].

It faces challenges related to security, compliance, privacy, and high availability [71].	It maintains low cost with maximizing efficiency and throughput [71]	It benefits by connecting with one cloud system and acquires its services, dedicated network, and increase transfer speed through protocol optimization [73].

RAN lacks centralize organization for emerging heterogeneous networks, flexibility to drift network services towards network verge for new application utilization and generate revenue from it [74].	Mostly faces two challenges such as scalability and cost effectiveness. Scalability depends on architectural design of DCN while cost depends on its power consumption [75].	

**Table 3:** Issues in current network forensics and MCC network forensics.

Issues	Current network forensics	MCC network forensics
Data acquisition [79]	No	Yes
Access to artifacts [79, 80]	No	Yes
Bandwidth utilization [73]	No	Yes
Chain of custody [14, 74, 75]	No	Yes
Data Integrity [74]	No	Yes
Privacy [81, 82]	No	Yes
Real time Analysis [74]	No	Yes
Volatile data [83, 84]	No	Yes
Forensics tools [14, 75, 79]	No	Yes

**Table 4:** Classification of network forensics frameworks.

Frameworks		Functions
Traceback	NFEA [71]	Proposes effective tracking range to provide admissible digital evidence with guarantee of integrity and authenticity of track data. Further, it marks packets at edge router which increase efficiency and decrease loss of data.
	LWIP [9]	Considers only time to live (TTL) field of IP header to trace out attack path in DDoS attacks. It used three algorithms that address three steps to make proposed scheme efficient, robust, and simple such as (a) embeds TTL value in IP header, (b) performed soon as DDoS attack occur, and (c) attack tree analysis algorithms is executed.
	Scalable NF [16]	Proposes scalable network forensics scheme for stealthy self-propagating attacks to traceback the origin of attack. Moreover, scheme is scalable in terms of computational time and space to accurately discover origin of attack. In addition, data reduction mechanism is used to identify deviations of each host and it acts as indication for a potential attack which is further process for forensics investigation.
	HB-SST [85]	Presents generic hopping based spread spectrum technique for network forensics traceback in anonymous communication networks. It provides randomized effect to mark network traffic in both time and frequency domains.
	ITP [8]	A protocol is design to traceback attacks in real time as well as periodically using compressed hash table in the router. Further, it addresses replay attacks through timestamp attached to the messages and its integrity is verified through using hash function. Moreover, it enhances detection rate of attacks by updating attack list periodically in routers.

Converged network	PBNF [87]	Proposes VoIP network forensics patterns that use to collect and analyze voice traffic in a systematic way.
	VoIP-NFDE [88]	A digital evidence procedure for VoIP network forensics is proposed especially for internet phone. Evidence is identified by comparing normal and abnormal packets in voice communication.
	VoIPEM [62]	Model based forensics method is proposed to identify malicious attacks in VoIP communication that formalize hypothesis through information gathering. Moreover, attack path is reconstructed by adapting secure temporal logic of action (S-TLA+) which provide clear evidence about attacks.

Intrusion detection system	AIDF [91]	An analytical intrusion detection framework proposed, based on probability model discovery approach & inference mechanism. It provides forensics explanation not only on intrusion alerts, but also on unidentified signature rules. Moreover, it integrates intrusion alerts from disseminated IDS sensors.
	DFITM [92]	Intrusion tolerance base dynamic forensics modeling is performed to enhance availability of forensics server in case of an attack. Modeling is conducted with finite state machine and forensics server availability is analyzed through numerical analysis.
	IIFDH [93]	Steganography is applied to identify alteration in log files performed by an intruder after his malicious attack. It maintains reliability and completeness of the evidence for future decisions.
	NFIDA [94]	Network forensics based on intrusion detection static and dynamic analysis is performed to provide complete record of data and logs while ensuring credibility and reliability.

Attack graphs	SA [95]	Proposes a framework that performs scalable analysis of attack scenarios by analyzing massive amount of alerts in real-time situation. Moreover, it also addresses individual attacks and its impacts on the enterprise.
	MLL-AT [96]	It identifies multistage network attacks and analyzes system risk by evaluating various security threads that occurs due to attack sequences.
	AGFE [97]	Integrates antiforensics mechanism with attack graph to fully observe intruders while deleting certain traces after attack performed.
	FCM [98]	Generate fuzzy cognitive map from attack graph with the help of genetic algorithm to find a worst attacks in the network. It simples a situation for network investigator to tackle such attacks with great concern.
	CSBH [17]	A probabilistic approach is proposed that integrates attack graph with hidden Markov model for exploring system states and its observation. It identifies the root cause of attack with providing automation, adaptability, and scalability in large network for cost benefit security hardens.
	AGVI [99]	RAVEN framework is proposed that reduces sophistication in large attack graphs by providing interactive visualize interfaces for user to illustrate attack graphs easily.

Distributive	ForNet [100]	Proposes distributive framework to collect network logs from different network devices in disseminated network. It analyze IP packet header for IP connection, ports, and various sessions through bloom filter tracking.
	DRNIFS [101]	It captures network packets soon as an attack is detected in a real-time situation. Moreover, it collects potential evidences that are deleted in most of the cases by intruders after its malicious attacks. It uses centralize network forensics server with disseminative detective agents.
	DCNFM [102]	Proposes framework that identifies potential risk, misbehavior of packets, and origin of attack with having distributed cooperative network forensics system. The system is comprised of client server architecture, with client agents installed on different system to capture network traffic logs from different network artifacts.
	DNF-IA [18]	It proposes artificial intelligence immunity theory to address network forensics in real time with keeping evidence in a safe way. It provides validity, integrality, and authenticity for evidence in a real time situation.

**Table 5:** Structure of network forensics frameworks.

Frameworks		Approach	Methods	Evaluation	Limitations	Performance
Traceback	NFEA [71]	LO, PM	Authenticated evidence marking scheme (AEMS)	Test bed & Simulation	Computational & Storage overhead	50% performance degrades when AEMS applied to each packet. However, performance gains 40% when it is applied to only select packets.
	LWIP [9]	PM	Lightweight IP traceback based on TTL	Tree analysis algorithm	Router overhead	Significant path reconstruction in DDoS attack
	Scalable-NF [16]	LO	Scalable network forensics	Real world traffic traces	Capture real time traffic	Reduce 97% of irrelevant data for analysis
	HB-SST [85]	Spread spectrum techniques	Hopping based spread spectrum	Simulation	Scalability	False positive decrease exponential with increase in signal length.
	ITP [8]	LO	IP traceback protocol (ITP)	Simulation	Router overhead	ITP shows better results in term of false positive rate & attack detection as comparing with existing frameworks

Converge network	PBNF [87]	LO	VoIP network forensics patterns	Suggest to use NFATs	Scalability, Forensics server bottle neck	Faster and structural investigation in VoIP traffic
	VoIP-NFDE [88]	LO	VoIP network forensics with digital evidence	Test bed	Time consuming, bandwidth utilization	Collects, analyzes, and performs forensics in VoIP DEFSOP operational stage
	VoIPEM [62]	LO	VoIP Evidence Model	S-TLC+	Not trace anonymous attacks	Identifies significant information relate to attacks

Intrusion detection system	AIDF [91]	Probabilistic model	Probabilistic discovery & inference	Test bed	Database for untreated data	Perfect discovery results in 16.67% and information combining from multiple IDS for forensics explanation is 87%
	DFITM [92]	Dynamic forensics intrusion tolerance	Formal methods	Finite state machine	Storage overhead	Enhancement of availability of forensics server with improvement of collected significant evidence
	IIFDH [93]	LO	Steganography	Prototyped	Scalability	Real-time detection with preservation of evidence
	NFIDA [94]	LO	Multi-dimensional analysis	Not applicable	Computational overhead	Records complete network data with providing data integrity that results in network forensics solution based on intrusion detection analysis.

Attack graph (AG)	SA [95]	Measure current & future attacks	Scalable analysis	Synthetic & real AG	Computational overhead	For large graph the integer value *k* increases when processing time increase. However it remains stable for small graphs
	MLL-AT [96]	Network attack modeling	Multi-level & layer attack tree	Case study	Scalability, Storage overhead	Model attack more accurately, address system risk
	AGFE [97]	Forensics examination	Anti-forensics injection in AG	Test bed	Scalability	Identifies alteration performed by intruders in log files.
	FCM [98]	Network security evaluation	finite cognitive map & genetic algorithm	Simulation	Observation depended, lack of awareness	Results best fit value of 1.64 that shows the probability of goal achieved.
	CSBH [17]	Probabilistic	Design model	Scenario based	Computational overhead	It finds that an approach is user centric, with complexity O (MN^2^).
	AGVI [99]	Visualization & Interaction	RAVEN	Not applicable	Visualization in real time situation	Address impact of HCI techniques on attack graphs

Distributive	ForNet [100]	distributive network forensics	Architecture	Not applicable	Limited attack detection due to lightweight filtering	Provide valuable, trustworthy information about network events
	DRNIFS [101]	LO, PM	Architecture	Not applicable	Storage overhead	Real time detection with quick incident response
	DCNFM [102]	LO	Client Server Architecture	Not applicable	Forensics server bottle neck, Storage overhead	Identifies origin of attack and potential risk
	DNF-IA [18]	LO	Dynamic network forensics model	Laboratory test	Lack of cryptography, forensics server bottle neck	Integrated, accurate results in real-time situation when attacks are occurred.

Approaches: LO: logging; PM: packet marking.

**Table 6:** Analysis of network forensics frameworks in context of adaptability to MCC.

Frameworks		Scalability	Overhead		Accuracy	Complexity	Privacy	Adaptability
			Computational	Storage				
Traceback	NFEA [71]	N/A	H	H	L	IM	L	N/A
	LWIP [9]	HT	H	M	M	AL	M	D
	Scalable NF [16]	VT	L	L	H	IV	L	M
	HB-SST [85]	HT	M	N/A	N/A	IM	N/A	D
	ITP [8]	N/A	H	H	M	IM	M	D

Converge networks	PBNF [87]	N/A	H	L	L	IM, AL	L	D
	VoIP-NFDE [88]	N/A	M	M	N/A	IM, CL, AL	L	D
	VoIPEM [62]	HT	M	M	N/A	IM, AL	L	N/A

Intrusion detection system	AIDF [91]	HT	M	M	N/A	IM, AL	N/A	D
	DFITM [92]	HT	H	L	N/A	IM, AL	N/A	D
	IIFDH [93]	N/A	H	L	N/A	AL	L	D
	NFIDA [94]	N/A	L	L	L	IM, AL	L	D

Attack graph	SA [95]	HT	M	L	N/A	AL	L	L
	MLL-AT [96]	N/A	M	L	N/A	IM, CL, AL	M	L
	AGFE [97]	N/A	H	M	L	IM, AL	M	D
	FCM [98]	HT	M	M	H	AL	M	M
	CSBH [17]	HT	L	L	M	AL	M	H
	AGVI [99]	HT	L	N/A	N/A	AL	H	M

Distribution	ForNet [100]	VT	M	M	N/A	CL, AL	L	M
	DRNIFS [101]	BT	M	M	N/A	AL	L	L
	DCNFM [102]	HT	M	M	L	CL, AL	L	L
	DNF-IA [18]	HT	L	L	L	IM, CL, AL	L	M

Scalability: HT: horizontal; VT: vertical; BT: both; N/A: not applicable.

Overhead: H: high; M: moderate; L: low; N/A: not applicable.

Accuracy: H: high; M: moderate; L: low; N/A: not applicable.

Complexity: IM: implementation; AL: analysis; CL: collection; IV: investigation.

Privacy: H: high; M: moderate; L: low; N/A: not applicable.

Adaptability: D: difficult; H: high; M: moderate; L: low; N/A: not applicable.

## List of Acronyms

3G-4G: Third-fourth generation
AGFE: Attack graph for forensic examination
AGVI: Attack graph visualization and interaction
AIDF: Analytical intrusion detection framework
CBSH: Cost-benefit security hardening
CFS: Collaborative forensics scheme
CSP: Cloud service provider
DCN: Data center network
DCNFM: Distributed cooperative network forensics model
DDoS: Distributed denial-of-service
DFITM: Dynamic forensics intrusion tolerance method
DNF-IA: Dynamical network forensics-immune agent
DoS: Denial-of-service
DRNIFS: Distributed real-time network intrusion forensics system
FaaS: Forensics-as-a-services
FCM: Fuzzy cognitive map
FTK: Forensics toolkit
GA: Genetic Algorithm
HB-SST: Hopping based spread spectrum techniques
HCI: Human computer interaction
I/O: Input/output
IDS: Intrusion detection system
IIFDH: Intrusion investigation framework data hiding
ITP: IP Traceback protocol
LTE: Long-term evaluation
LWIP: Lightweight IP traceback
MCC: Mobile cloud computing
MLL-AT: Multilevel and -layer attack tree
NFAT: Network forensics analysis tool
NFEA: Network forensics evidence acquisition
NFF: Network forensics framework
NFI: Network forensics investigator
NIST: National Institute of Standards & Technology
PBNF: Pattern based network forensics
RAN: Radio access network
QoS: Quality of service
RMW: Random moonwalk
SA: Scalable analysis
SLA: Service level agreement
S-TLA+: Secure Temporal logic of action
TDPM: Topology assisted deterministic packet marking
TTL: Time-to-live
VoIP: Voice over Internet protocol
VoIPEM: Voice over Internet protocol evidence model
VoIP-NFDE: VoIP-network forensic with digital evidence
Wi-Fi: Wireless fidelity
WLAN: Wireless local area network.
